# Responses to Pandemic (H1N1) 2009, Australia

**DOI:** 10.3201/eid1608.100132

**Published:** 2010-08

**Authors:** Keith Eastwood, David N. Durrheim, Michelle Butler, Alison Jones

**Affiliations:** Hunter New England Health, Newcastle, New South Wales, Australia (K. Eastwood, D.N. Durrheim, M. Butler); University of Newcastle, Newcastle (D.N. Durrheim); University of Western Sydney, Sydney, New South Wales, Australia (A. Jones)

**Keywords:** Pandemic, influenza, H1N1 subtype, perception, health knowledge, attitude, practice, Australia, viruses, research

## Abstract

In 2007, adults in Australia were interviewed about their willingness to comply with potential health interventions during a hypothetical influenza outbreak. After the first wave of pandemic (H1N1) 2009 in Australia, many of the same respondents were interviewed about behavior and protection measures they actually adopted. Of the original 1,155 respondents, follow-up interviews were conducted for 830 (71.9%). Overall, 20.4% of respondents in 2009 had recently experienced influenza-like illness, 77.7% perceived pandemic (H1N1) 2009 to be mild, and 77.8% reported low anxiety. Only 14.5% could correctly answer 4 questions about influenza virus transmission, symptoms, and infection control. Some reported increasing handwashing (46.6%) and covering coughs and sneezes (27.8%) to reduce transmission. Compared with intentions reported in 2007, stated compliance with quarantine or isolation measures in 2009 remained high. However, only respondents who perceived pandemic (H1N1) 2009 as serious or who had attained higher educational levels expressed intention to comply with social distancing measures.

## Abstract 2

Perception of risk affects compliance with public health control measures.

The World Health Organization (WHO) declared a public health event of international importance on April 24, 2009, after recognition of a novel pandemic influenza virus strain, pH1N1, now called pandemic (H1N1) 2009 virus, which caused serious disease and deaths in Mexico and other parts of North America. This declaration triggered an immediate response in Australia; national pandemic plans were implemented, and the public was alerted to the risk and the activities that could keep them from contracting and spreading the infection.

Imported cases of pandemic (H1N1) 2009 were first identified in Australia on May 7, 2009, and within a month, local transmission had been identified in all 8 states and territories (*1**,**2*). By September 1, 2009, of Australia’s population of 22 million, 154 had died and 4,440 had been hospitalized for pandemic (H1N1) 2009 (*3*). Within the first 2 months of the outbreak, the Australian Commonwealth instituted 3 management phases: delay, contain, and protect. Each phase required different messages to the public and healthcare workers (*1**,**2**,**4*). Ensuring consistent implementation through Australia’s 3 government levels—national, state, and local—was challenging. The delay phase was aimed at preventing pandemic (H1N1) 2009 from arriving in Australia and focused attention on border control and communication with international travelers. However, after local transmission was recognized and the disease became established in Australia, the contain phase was implemented with an emphasis on identifying cases and tracing contacts. Those with confirmed pandemic (H1N1) 2009 infection and their contacts were actively managed by using isolation, home quarantine, antiviral medication, and enhanced infection control practices to reduce the spread of disease. Finally, when it became clear that pandemic (H1N1) 2009 infection in Australia was less severe than initially considered and that the workload was adversely affecting the provision of health services, the protect phase was implemented and the public health response was changed to early detection and management of infection in persons from recognized risk groups. The change in focus (from aggressively tracking new infections to treating all persons in Australia with influenza-like illness [ILI] to concentrating on those in high-risk groups) presented a major risk-communication challenge for health authorities.

The success of the pandemic management plan in Australia depends critically on public compliance with health measures (*5**,**6*). A study completed in 2007 found that a high proportion of respondents reported willingness to accept a range of public health measures (although the scenario provided in that study was a more severe pandemic) (*7*). In that study, 1,166 (58.0%) of 2,012 adults contacted participated in the survey. Nearly all (1,155) agreed to be available for future related research.

The 2009 pandemic provided a unique opportunity to conduct a follow-up study to compare respondents’ previously reported willingness to adopt public health measures with their experiences during and after pandemic (H1N1) 2009 in Australia. We thus conducted a study during the protect phase, from August 20 through September 11, 2009, almost 4 months after the WHO declaration and 1 month after the peak of reported hospitalizations from the first wave of pandemic (H1N1) 2009 in Australia (*1**,**2**,**4*). We sought to identify the level of public knowledge concerning measures required to contain pandemic influenza spread, social impact of the pandemic wave, effectiveness of communication, compliance with control measures instituted by public health authorities, and relationships among these parameters.

## Methods

### Study Protocol and Participants

In the original 2007 study, the sample was selected in a randomized manner from printed telephone directories (for 2007) by using a quota system that ensured good representation from across the country. Eligible persons were >18 years of age, could converse in English, and provided verbal consent. Thus, in the current (2009) study, the youngest possible participant was 20 years of age.

Of the 1,155 participants in the 2007 study who had agreed to be involved in future research, 43 were excluded from the 2009 study because they had died, were unable to communicate, or had moved and were untraceable; 197 were not reachable at their recorded telephone number; and 85 refused to participate. Thus, 830 (71.9%) persons were successfully interviewed in the 2009 study. Ethics approval was obtained from the University of Newcastle’s Human Research Ethics Committee (approval no. H-2009–0288).

In each study, data were collected by computer-assisted telephone interview, and an introductory letter was sent to households a week before telephone contact was attempted. Interviews took an average of 14 minutes to complete and were conducted on weekdays and Saturdays between 9:00 am and 8:00 pm local time. The surveys were conducted by professional telephone interviewers who had extensive experience collecting public health data. A rigorous training program, which included dummy interviewing and written interviewing protocols, encouraged a consistent approach. As many as 10 attempts were made to contact each person in the database. The script for the compliance questions was identical to that used in 2007, but other questions were tailored to the new pandemic. In the survey, we chose to identify the novel influenza disease as “swine flu” because this was the term most commonly used in the media in Australia at the time of study and was accepted by the public.

### Scope of Interview

Interviewers asked structured questions related to respondents’ recent experience with pandemic (H1N1) 2009. Knowledge questions asked about pandemic (H1N1) 2009 transmission, its symptoms, and infection control; and 1 question gauged comprehension of the global situation. A variety of questions assessed anxiety, impact, behavior, compliance with public health advice, and access to and perceived success of communications. The interviews included closed- and open-ended questions. Questions regarding knowledge provided true or false response options; the situational awareness question was multiple choice with 3 options; questions about impact were answered on a scale of 4 levels (nil, little, quite, and extremely); and questions about perceptions encouraged open-ended answers that were subsequently coded. To assist implementation of a national vaccination program in Australia, we also investigated respondents’ willingness to accept pandemic (H1N1) 2009 influenza vaccine and specific related concerns; these results are reported elsewhere (*8*).

### Statistical Analyses

Analyses were conducted by using base SAS and SAS/STAT components of SAS 9.13 statistical software (SAS Institute Inc, Cary, NC, USA). Odds ratios and χ^2^ tests were used to look for significant associations between sex, age group, perception of severity and educational status, and willingness to comply with public health interventions. A stepwise multivariate analysis that included variables of statistical significance was used. The sample was weighted to the age–sex distribution of the adult population of Australia by using June 2008 data projections (*9*).

## Results

Demographics for the 830 respondents closely resembled those of the resident population of Australia during June 2008. Demographics for the 2009 study participants did not differ significantly from those of the 2007 study participants (*7*). Among the 2009 study population, 75.7% lived in urban areas, 20.8% in rural areas, and 3.3% in remote areas. Women (62.3%) and older age groups were moderately overrepresented. More information on the sample demographics is available from the earlier study report (*7*).

### Knowledge

We asked 4 questions about knowledge of pandemic (H1N1) 2009 transmission, symptoms, and infection control measures: 1) almost everyone (99.4%, 825/830) knew that “handwashing and using a tissue to cover your mouth when coughing are practical ways of reducing the spread of flu,” 2) 17.2% (143/830) were unaware that “swine flu spreads very easily in the community,” 3) 44.9% (373/830) incorrectly considered that “cough and rash are typical of swine flu,” and 4) 30.2% (251/830) incorrectly reported that “swine flu never seriously affects people who have good health.” Overall, only 14.5% (120/830) answered all 4 questions correctly, 48.9% (406/830) answered 3 correctly, and 30.8% (256/830) answered 2 correctly.

Pandemic (H1N1) 2009 situational awareness was investigated with a question aimed at determining how well the Australian public understood the extent of the outbreak. At the beginning of the study period, WHO had reported 177,457 confirmed cases from 170 countries and territories and noted that this understated the true number because testing had ceased in many countries (*10*). Only 56.5% (469/830) appreciated that there had been >100,000 cases around the world at the time of the study; 24.1% (200/830) answered that there had only “been ≈10,000 cases mainly affecting people in Mexico, the United States and the United Kingdom”; 8.1% (67/830) indicated that there had “been ≈10,000 swine flu cases mainly reported from Australia”; 11.2%, 93/830 reported that they did not know how many cases there had been; and 1 refused to answer.

### Impact

Having experienced ILI (“fever, cough and tiredness”) during the pandemic period was reported by 20.4% (169/830) of respondents; of these, 8.1% (67/830) had obtained a medical diagnosis but only 0.2% (2/830) had had their condition confirmed as pandemic (H1N1) 2009 by laboratory testing. The average duration of ILI was 9.2 days (median 6 days, interquartile range 4–10 days).

Most (77.7%, 645/830) respondents perceived pandemic (H1N1) 2009 as a mild disease or an only occasionally severe disease, 20.2% (168/830) considered it either mostly or always severe, and 2.0% (17/830) did not know. Most (77.8%, 646/830) reported being only a little or not concerned that they or a member of their family may become infected, 5.3% (44/830) were extremely concerned, and 16.9% (140/830) were quite concerned. In terms of risk perception, 25.4% (211/830) of respondents considered themselves to be in a group at risk for more severe illness or higher likelihood of infection. In terms of disruption as a result of public health containment measures enacted during the containment phase, most (94.5%, 784**/**830) respondents experienced no or only minor disruption, 4.0% (33/830) moderate disruption, 1.2% (10/830) major disruption, and 0.3% (3/830) were unsure of the effect these measures had on their lives.

### Personal Protection

Respondents described specific behavioral changes that they had adopted to reduce the transmission of pandemic (H1N1) 2009. Increased handwashing was reported by 46.6% (387/830) and covering coughs and sneezes by 27.8% (231/830). Only 8.7% (72/830) had purchased masks, 6.0% (50/830) reported having worn a mask in public, 3.3% (27/830) said they had “purchased (not just been prescribed) an antiviral drug such as Tamiflu or Relenza,” and 12.4% (103/830) indicated that they had “spent more time than usual cleaning the house.”

### Compliance with Public Health Containment Measures

The 2007 study used a scenario describing a future influenza pandemic as a potentially severe event. The interviewer then asked questions about willingness to comply with a range of public health containment measures. Although compliance with public health requests for self and community quarantine measures remained high during the 2009 survey, stated compliance with key social distancing activities significantly decreased (Table 1).

**Table 1:** Adults’ reported willingness to comply with public health authority requests with regard to influenza pandemic, Australia*

Public health request	% (no.) willing to comply†		OR (95% CI)	p value
	2007, n = 1,166	2009, n = 830		
Home quarantine for 1 wk if exposed	97.5 (1,137)	96.0 (797)	1.62 (0.95–2.80)	0.059
Local quarantine of an affected area	95.2 (1,103)‡	94.6 (785)	1.13 (0.74–1.72)	0.555
Avoid public events for 1 mo	98.3 (1,146)	82.8 (687)	11.93 (7.35–20.28)	<0.001
Avoid social gatherings for 1 mo	97.2 (1,133)	62.7 (520)	20.47 (14.01–30.67)	<0.001
Wear a surgical mask in public	95.1 (1,109)	72.4 (601)	7.41 (5.42–10.25)	<0.001

*OR, odds ratio; CI, confidence interval. †2007 responses were to hypothetical pandemic; 2009 responses were to actual pandemic (H1N1) 2009. ‡Because responses were not collected from 7 respondents, n = 1,159.

Compliance was analyzed by age group, gender, highest educational level achieved, experience of ILI in the past 3 months, stated degree of concern, performance on knowledge questions, and self-determined risk group. All factors were included in a multivariate logistical regression model; statistically significant findings are shown in Table 2.

**Table 2:** Predicted compliance with public health authority requests with regard to influenza pandemic, Australia, 2009*

Variable	Home quarantine for 1 wk if exposed			Local quarantine of an affected area			Avoid public events for 1 mo			Avoid social gatherings for 1 mo			Wear a surgical mask in public	
OR (95% CI)	p value	OR (95% CI)	p value	OR (95% CI)	p value	OR (95% CI)	p value		OR (95% CI)	p value
Sex														
F	2.34 (1.21–4.53)	0.012												
M	1.00													
Age range, y														
20–40	0.30 (0.10–0.89)	0.030		0.29 (0.12–0.74)	0.010		0.98 (0.58–1.65)	0.930					0.56 (0.37–0.87)	0.009
41–60	0.37 (0.12–1.12)	0.078		0.34 (0.13–0.87)	0.024		0.57 (0.35–0.95)	0.031					0.58 (0.37–0.90)	0.014
>61	1.00			1.00			1.00						1.00	
Personally experienced ILI during pandemic														
Yes				6.94 (2.10–22.95)	0.001								1.70 (1.09–2.64)	0.019
No				1.00									1.00	
Concerned														
Quite/ extremely							4.72 (2.60–8.54)	<0.001		2.63 (1.75–3.95)	<0.001		3.31 (2.12–5.18)	<0.001
A little							2.82 (1.86–4.29)	<0.001		1.49 (1.09–2.06)	0.014		2.85 (2.00–4.04)	<0.001
Not							1.00			1.00			1.00	
Highest education level														
Tertiary†							1.82 (1.23–2.68)	0.003					1.88 (1.37–2.59)	0.001
Other							1.00						1.00	

*Multivariate logistic regression analysis for 830 respondents, sample weighted to the age and sex distribution of the population of Australia (*9*). Only significant results are shown. OR, odds ratio; CI, confidence interval; ILI, influenza-like illness. †University or professional qualifications.

Women were significantly more likely to agree to home quarantine if requested by public health officials. Those in the oldest age group (>61 years) and those who had experienced ILI were also more likely to agree to local quarantine, such as remaining within town limits when high influenza activity is evident. Perceptions of anxiety and level of education were associated with willingness to avoid public events; level of anxiety was also associated with willingness to avoid social gatherings and with wearing a mask to control influenza.

### Communication

Slightly more than one third (288/830) of respondents reported that they had sought information on pandemic (H1N1) 2009 during the first pandemic wave in Australia. The most common sources were general practitioners (19.3%, 160/830), other healthcare workers (9.5%, 79/830), and government websites (13.3%, 110/830). The public health department was contacted by 4.8% (40/830), and the national health hotline was used by only 3.1% (26/830). Most (61.6%, 511/830) respondents reported not “actively searching for news on swine flu in the media,” although 7.8% (65/830) sought a daily update.

Most (69.3%, 575/830) respondents thought that “health authorities had provided sufficient information on swine flu,” 18.1% (150/830, including 12 who replied that they had not seen or heard any information) reported that there had not been enough information, 9.9% (82/830) reported that there had been too much information, and 2.8% (23/830) were unsure. To assess whether the media campaign had been effective, we asked the 818 who had received media information whether it had changed any of their behavior. More than half the respondents (53.9%, 441/818) reported having “paid more attention to covering coughs and sneezes,” 47.1% (385/818) had “increased the frequency of handwashing,” and 43.8% (358/818) had “stayed at home when sick to reduce spreading the disease.”

During the protect phase, which was declared on June 17, 2009, the Australian Commonwealth Government conducted a national information campaign (paid television, radio, and print advertisements). To determine the effect of this media campaign, which had been conducted 2 months after the onset of the emergency and after local and state messages had been broadcast, we asked participants whether they had seen or heard any of the advertisements and whether these had specifically prompted any change in their behavior. We found that 19.5% (162/830) of respondents neither heard nor saw any of these media messages, 14.3% (119/830) had noticed them every day, 44.1% (366/830) noticed them about once a week, 19.2% (159/830) noticed them about once a month, and 2.9% (24/830) were unsure how often they had noticed them. Of those who had seen advertisements, 88.2% (568/644) said that the information had little or no effect on their behavior. Multivariate analysis indicated that awareness of the information provided in the Commonwealth's promotional campaign was not associated with willingness to comply with public health containment measures. When we asked whether “health authorities should post hygiene messages at bus terminals, train stations and airports, such as avoiding travel when sick, and covering coughs and sneezes,” 95.4% (792/830) of respondents answered affirmatively.

## Discussion

It is widely accepted that the first wave of the 2009 influenza pandemic in Australia resulted mostly in a relatively mild disease with little of the forecasted social and economic consequences factored into prepandemic planning or relayed in prepandemic media messages (*1**,**2**,**4**,**11*). However, valuable lessons can be learned from the management of this pandemic and applied in the future. Public perceptions formed during the pandemic wave may influence future responses to public health disasters and must be considered when preparing future risk communication strategies.

Despite the considerable media attention to the pandemic, a high proportion of adults in Australia poorly understood the fundamental aspects of transmission, symptoms, and impact. Most persons did not actively seek information on pandemic (H1N1) 2009, but when they did seek information, they were most likely to turn to a general practitioner or other healthcare worker or a government website rather than use the national health hotline that was set up to ease the pressure on health professionals. This finding is in contrast with use of the UK call center, “NHS Direct,” which experienced a heavy load of calls (*12*). There is clearly a need to improve basic health literacy through educational initiatives in schools, public health awareness campaigns, and other creative methods, and to more effectively channel enquiries away from those working on the front lines during emergencies.

We found that stated willingness to comply with social distancing requests decreased significantly in 2009 compared with 2007. However, multivariate analysis indicated that acceptance of public health containment measures was statistically more likely among those experiencing a higher level of concern, an observation supported by other researchers (*13*). The reduced level of compliance reported in the 2009 study likely resulted from the perceived mildness of disease and may result in less cooperation during future pandemic waves or other health emergencies. However, translating risk perception into behavior change is challenging (*14*). In a world constantly threatened by emerging infectious diseases, promoting effective risk communication strategies that accurately inform the public and health professionals of appropriate behavior changes that can be made to mitigate personal and community risk is essential (*15*). In addition, interview responses suggested a need for a closer tailoring of risk communication mechanisms and messages to adequately inform person’s responses under the stress of emergency conditions (*16**,**17*).

Respondents may have considered pandemic (H1N1) 2009 to be a mild disease because the perceived focus of health officials was on simple containment measures such as increased handwashing and covering sneezes rather than sophisticated approaches, which may have created an impression of a trivial threat. Perception of risk governs the level of response; thus, the appropriate risk level must be communicated, although difficult to achieve particularly in the early stages when epidemiologic data are lacking (*17**–**19*). During a health emergency such as a pandemic, all levels of the health system must ensure that consistent messages are relayed to the public and clearly explain the value of proposed interventions.

Because 20% of respondents reported that they had not seen any of the advertisements during the multimedia blitz and 88% of those who did see them claimed that the advertising had little or no effect on their behavior, we conclude that the intensive media promotion did not substantially influence infection control behavior. It is possible that those who were likely to change their behavior had already done so as a result of earlier messages delivered through media statements from local health officials. This possibility supports findings from the 2007 study that indicated that persons seem to place most trust in state or regional health representatives for their disaster health information rather than national identities; thus, greater emphasis on local messaging may be merited (*7*).

Compared with many parts of the world, Australia is relatively sparsely populated; the preponderance of the population live in large cities and towns on the Eastern Seaboard. We ensured that the study population included a representative cross-section of the adult population of Australia that closely matched the most recently available census information (*9*); persons from inland rural and remote areas were proportionally represented. Women and elderly persons were slightly overrepresented, in keeping with other national computer-assisted telephone interview populations in Australia. To some extent our analysis accounted for this overrepresentation by weighting to the latest population estimates of age and gender. Because the study design was based on telephone contact, only persons with a landline telephone were included in the study. In Australia, landline telephone coverage is generally high (*20**,**21*), but our sample likely underrepresents disadvantaged groups such as indigenous Australians, particularly those who may live in remote communities without landline coverage, and persons with lower incomes who cannot afford a telephone. In addition, children were not included in the survey, yet their knowledge and behaviors may prove valuable in educating adults on preparedness and response (*22*). Those who declined to be interviewed or were excluded because of language and comprehension problems are likely to be more difficult to reach with conventional communication methods, further emphasizing the value of novel and enhanced communication strategies appealing to a broader demographic spectrum.

The gap between what health officials require the public to do in a pandemic and what members of the public are prepared to do seems to be growing. Our findings suggest that discordance between what people say they will do and what they actually do is related to their perception of risk. The public should be equipped with the appropriate knowledge and skills to positively influence their attitudes and behavior during a future pandemic wave or communicable disease event and to enable them to better interpret broadcasted risk assessments. Such a literacy program would be useful for pandemic preparedness, generating appropriate reassurance or concern, and could potentially achieve broader health goals.
